# Dynamics of the impacts of *Pratylenchus penetrans* on Gisela® cherry rootstocks

**DOI:** 10.21307/jofnem-2019-008

**Published:** 2019-04-15

**Authors:** Thomas Forge, Denise Neilsen, Gerry Neilsen, Suzanne Blatt

**Affiliations:** 1 Agriculture and Agri-Food Canada, 4200 Hwy 97, Summerland, BC V0H 1Z0, Canada; 2 Agriculture and Agri-Food Canada, 32 Main Street, Kentville, NS B4N 1J5, Canada

**Keywords:** Lesion nematode, *Pratylenchus penetrans*, Rootstock, Orchard, Host-parasite relationship, Plant disease loss

## Abstract

Sweet cherry growers are increasingly using semi-dwarfing rootstocks, including the Gisela® series, when replanting orchards. Little is known of the susceptibility of these new cherry rootstocks to *Pratylenchus penetrans*, a recognized pest of temperate fruit trees worldwide. Two field experiments were planted in 2010, one in the Okanagan Valley of British Columbia and one in the Annapolis Valley of Nova Scotia. Each experiment was a factorial combination of three rootstocks (Gi.3, Gi.5, and Gi.6) × three training systems, with six replicate four-tree plots of each of the nine combinations. Both sites were fumigated prior to planting and population densities of *P. penetrans* in roots and root-zone soil were subsequently monitored from 2013 through 2017. None of the *P. penetrans* population parameters (nematodes/kg soil, nematodes/g fine root, and nematodes/kg soil including roots) differed among rootstocks at either site, suggesting that the rootstocks did not differ in their ability to host *P. penetrans*. At the British Columbia site only there was an inverse relationship between *P. penetrans* population densities and tree size for Gi.3 trees in four years and for Gi.6 in 2017, suggesting that Gi.3 rootstock is less tolerant than Gi.5 and Gi.6 rootstocks.

Sweet cherry production is growing rapidly in North America, particularly in the Pacific Northwest and British Columbia. As old orchards are being renovated, growers are shifting to higher density plantings using semi-dwarfing rootstocks such as the Gisela® series (*P. cerasus* L. × *P. canascens* L.). The root-lesion nematode, *Pratylenchus penetrans* (Cobb) Filipjev and Schuurmans Stekhoven, is recognized as an important pest of fruit trees, including sweet cherry grown in temperate regions (Edgerton and Parker, 1958; Mai et al., 1994; Melakeberhan et al., 1994, 1997, 2000). Relatively little is known, however, of the relative susceptibility or tolerance of the new semi-dwarfing cherry rootstocks to *P. penetrans* or any other plant-parasitic nematodes (Franken-Bembenek, 2008). Comparing *P. penetrans* inoculated to non-inoculated non-grafted seedlings under greenhouse conditions, Melakeberhan et al. (1994, 1997) demonstrated that growth of Gisela 6 (previously GI-148-1) and Gisela 7 (previously GI-148-8) rootstocks was reduced by *P. penetrans*, particularly when the trees were grown under nutrient-poor conditions.

Current understanding of the effects of *P. penetrans* on fruit trees has primarily been the result of controlled-inoculation experiments comparing growth of juvenile, non-fruit bearing trees planted in either inoculated and non-inoculated greenhouse pots (Johnson et al., 1978; Mai et al., 1994; Melakeberhan et al., 1994, 1997, 2000), or in fumigated and non-fumigated field soil (Edgerton and Parker, 1958; Olthoff et al., 1989; Mai et al., 1994). Data relating *P. penetrans* populations to the vigor or yield of established, fruit bearing trees are limited, although such data would be valuable for illustrating the potential benefits of targeted nematode management in producing orchards. Detection and interpretation of relationships between nematode population densities and the vigor or yield of established trees, or any other perennial crop, under field conditions is complicated by the multitude of other factors that can impinge on tree growth and obscure nematode effects. Significant year-to-year variation in nematode population dynamics may or may not coincide with inter-annual variation in other factors affecting tree growth, and nematode damage occurring in one year can affect bud set and storage of carbohydrates and other resources, thereby affecting growth and yield in subsequent years (Schreiner et al., 2012). Consequently, the impacts of nematode feeding on established fruit trees can be viewed as accumulating over multiple years, with observable impacts varying from year-to-year as nematode population densities change along with other factors affecting tree growth.

The goal of our study was to compare the susceptibility of Gisela 3 (Gi.3), Gisela 5 (Gi.5), and Gisela 6 (Gi.6) cherry rootstocks under field conditions. Our specific objectives were to (i) determine if population densities of *P. penetrans* developing over multiple years in roots and root-zone soil differ among the rootstocks; and (ii) assess relationships between *P. penetrans* population densities and tree growth and yield, over multiple years.

## Materials and methods

### Experimental design

Two identical field experiments were planted in 2010, one in the Okanagan Valley of British Columbia and one in the Annapolis Valley of Nova Scotia, as part of a continent-wide series of trials (http://www.nc140.org; Neilsen et al., 2016). The British Columbia site was planted on an Agur Lake loamy sand (Wittneben, 1986) on the grounds of the Agriculture and Agri-Food Canada Summerland Research and Development Centre (49.6006°N, 119.6778°W). The Nova Scotia site was planted on a Berwick sandy loam on the grounds of the AAFC Kentville Research and Development Centre (45.057871°N, 64.484166°W). The British Columbia site was fumigated prior to planting using Vapam® at label rate and the Nova Scotia site was fumigated using Telone® at the label rate. Both experiments were established as a factorial combination of three rootstocks (Gi.3, Gi.5, and Gi.6) × three training systems. The training systems were either an “axe” system (Tall Spindle Axe), a bush system (Kym Greene Bush), or a planar system with the tree planted at 45° and the main stem trained horizontally to support a number of upright limbs (Upright Fruiting Offshoots). All trees were grafted to the variety “Skeena” and planted at both sites with a spacing of 1.5 m between trees and 4 m between rows. In British Columbia, there were six replicate four-tree plots of each of the nine combinations of rootstock and training system, arranged in a randomized complete block design, with one plot of each of the nine combinations represented in each of six rows which constituted blocks. The four trees in each plot were comprised of two guard trees on each side of two measurement trees. In Nova Scotia, trees were established in four rows with two blocks per row and with each block comprised of one three-tree plot of each of the nine combinations of rootstock and training system. Plots were randomized using a split-plot design where rootstock was randomized within training system and training system was randomized within the block. The three trees in each plot comprised of two measurement trees and a single guard tree.

In British Columbia, the trees were irrigated daily from approximately mid-April through September through two drip lines that ran down both sides of each row, positioned approximately 30 cm out from and parallel to the tree row. Each line had drip emitters (2 L hr^−1^ flow rate drip line) located at 30-cm intervals down along each irrigation line. Water was applied each day to supply 100% of the estimated water lost to evapotranspiration (ET) the previous day; an electronic atmometer (ETGage, Loveland, CO) was used to measure base ET which was adjusted to estimated ET for a cherry orchard using a crop coefficient curve (Allen et al., 1998). Nutrients were supplied each spring through fertigation, with a total of 30, 20, 20, and 0.17 g N, P, K, and B applied per tree starting immediately after bloom: all of the P was applied in 1 d, K and B were applied over a 4 wk period, and N was applied over a 6 wk period. A 1 m wide strip on either side of the tree row was treated with herbicides regularly to minimize competing vegetation (British Columbia Tree Fruit Production Guide; www.bctfpg.ca; accessed November 9, 2018). Foliar pest control measures were implemented according to standard production practices (British Columbia Tree Fruit Production Guide; www.bctfpg.ca; accessed November 9, 2018). At the Nova Scotia site, 17-17-17 fertilizer was broadcast-applied each spring at 200 kg ha^−1^, and lime was applied in 2009, 2012, and 2016. The trees were blossom thinned, and no fruit were harvested in the 2011 and 2012 growing seasons.

### Sampling and analyses

Beginning in 2013, the first year of fruit production, composite samples of root-zone soil were taken from each plot of the British Columbia experiment in April (2013) or June (subsequent years, before cherry harvest) and August (post-harvest) of each year through 2017. The Nova Scotia site was similarly sampled post-harvest in October of 2015, 2016, and 2017. Each sample was a composite of three 2 cm diameter × 30 cm deep cores taken from around the base of each of the two measurement trees in each plot. The cores were collected approximately 30 cm from the trunk of each tree at approximately 0, 45, and 90 degrees out from the row axis.

In the laboratory, fresh soil samples were hand-mixed and passed through a 6 mm sieve to remove stones and root fragments. Nematodes were extracted from 100 ml subsamples using a modified wet sieving-sucrose centrifugation technique (Forge and Kimpinski, 2007). Root fragments collected from each soil sample were separated into >2 mm and <2 mm diameter size classes. The <2 mm diameter root fragments were chopped into 1 to 2 cm long pieces, washed over a 250 µm sieve with a stream of tap water, and subjected to root-lesion nematode extraction over 7 d using a shaker method (Ingham, 1994). After root nematode extraction, coarse and fine root samples were air-dried and weighed. Root biomass data were expressed and analyzed as g fine roots per kg soil and g coarse roots per kg soil. Counts of *P. penetrans* extracted from soil were expressed and analyzed on a per kg soil basis. Counts of *P. penetrans* extracted from roots were expressed and analyzed as *P. penetrans* per g root. The total number of *P. penetrans* per kg soil, roots inclusive, was calculated for each sample by multiplying the biomass of fine roots per kg soil by the number of *P. penetrans* per g root, and adding the value to the number of *P. penetrans* per kg soil.

Tree trunk diameters were measured in two perpendicular directions at the end of each growing season at 0.3 m above the graft union; in Nova Scotia, trunk circumferences rather than diameters were measured. Trunk cross-sectional areas were calculated for each measurement tree. At harvest, individual tree yields were measured and mean fruit mass was assessed on a random sample of 100 fruit. Leaf stomatal conductance was measured periodically using a LiCor 1600 porometer (LiCor, Lincoln, NE, USA) between 11:00 and 13:00 hr on fully expanded leaves exposed to sun. Midday stem water potentials were measured bi-weekly using a pressure chamber (PMS Instrument Co., Corvallis, OR, USA) on leaves that were previously covered with black plastic and aluminum foil according to McCutchan and Shackel (1992).

### Data analyses

*Pratylenchus penetrans* population parameters (nematodes per kg soil, nematodes per g fine root, and nematodes per kg soil including roots) and root biomass data were subjected to mixed-model two-way repeated measures analysis of variance using Proc MIXED in SAS (Statistical Analysis Systems, Cary, NC). Fixed factors were rootstock, training system, and their interaction. Block was treated as a random factor in the model and sample date was considered in the model as repeated measures. Final analyses were conducted on log-transformed data to minimize heteroscedasticity and improve model fit. For the British Columbia site, which had two sample dates per year, these analyses were first conducted with the data separated by individual sample dates and subsequently with year as a factor in which the two individual sample dates were nested.

The influences of *P. penetrans* populations on tree water relations (mean annual stem water potential; minimum annual stem water potential; mean annual stomatal conductance) and tree growth parameters (trunk cross-sectional area; fruit yield; fine root biomass; total root biomass) were determined using analysis of covariance. Thus, for each year, simple and interactive effects of the *P. penetrans* population parameters (co-variates) and rootstock and training system (fixed factors) on tree water relations and growth parameters were assessed using Proc MIXED in SAS. These analyses were conducted using regular annual *P. penetrans* population data, and also using *P. penetrans* population parameters that were calculated for each year after the first as a running average, to reflect multi-year cumulative nematode impacts. Initial analyses indicated significant rootstock × *P. penetrans* interactions; consequently, and because the three rootstocks are already well known to affect overall tree growth (http://www.nc140.org; Neilsen et al., 2016), the final ANCOVA model assessed single and interactive effects of *P. penetrans* and training system, with separate analyses for each combination of rootstock and year.

## Results

**Figure 1:**
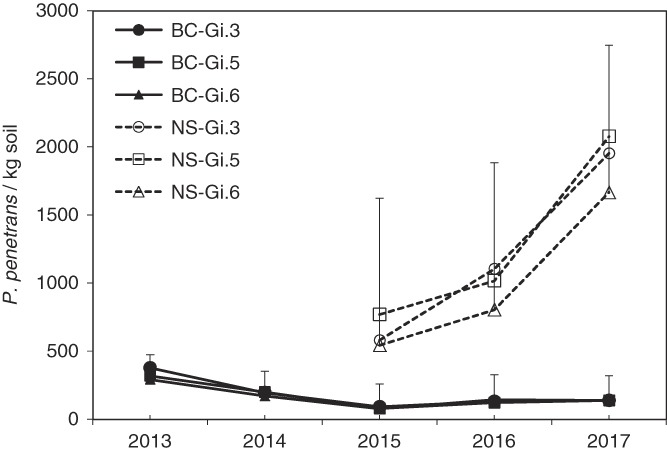
Population densities of *Pratylenchus penetrans* (per kg soil, roots inclusive) under Gi.3, Gi.5, and Gi.6 trees during the course of the study, at the Summerland, British Columbia (BC, solid lines) and Kentville, Nova Scotia (NS, dashed lines) sites. Error bars represent one standard deviation above the minimum value at each date.

For both sites and all nematode population parameters, sample date had a significant main-factor effect but there were no significant interactions between sample date and any of the fixed factors. Population densities of *P. penetrans* remained relatively low over the five years of sampling at the British Columbia site (Fig. 1). In contrast, *P. penetrans* population densities in Nova Scotia were overall almost 10× larger than in British Columbia and increased over the three years of sampling (Fig. 1).

### Effects of rootstocks and training systems on *P. penetrans* population densities and roots

In British Columbia, there tended (*p* = 0.077) to be main-factor effect of rootstock on *P. penetrans* per g root, with Gi.3 supporting greater population densities than Gi.5, with Gi.6 being intermediate (Table 1). There was no significant effect of rootstock or rootstock × sample date interaction on any other nematode population parameter at the British Columbia site, or on any parameter at the Nova Scotia site.

**Table 1:** Main-factor effects (averaged over sample dates) of Gisela 3 (Gi.3), Gisela 5 (Gi.5), and Gisela 6 (Gi.6) rootstocks and training systems (KGB, Kym Greene Bush; TSA, Tall Spindle Axe; UFO, Upright Fruiting Offshoot) on *P. penetrans* population densities in soil alone (Pp/kg soil), roots (Pp/g dry root), and soil and roots combined (Pp/kg soil with roots), at Summerland, British Columbia, and Kentville, Nova Scotia sites. For both sites and all nematode population parameters, sample date had a significant main-factor effect but there were no significant sample date × fixed factor interactions. Values labelled with different letters are significantly different (paired t-tests, p < 0.05).

	Summerland, BC			Kentville, NS		
	Pp/kg soil	Pp/g root	Pp/kg soil with roots	Pp/kg soil	Pp/g root	Pp/kg soil with roots
*Rootstock (RS)*						
Gi.3	140	104a	169	960	507	1,149
Gi.5	139	67b	153	1,029	551	1,239
Gi.6	127	81ab	148	829	398	958
*Training system (TS)*						
KGB	131b	91	150b	947	470b	1,106
TSA	121c	73	151b	1,079	624a	1,299
UFO	153a	89	168a	792	359b	935
*ANOVA P-values*						
RS	0.871	0.077	0.21	0.743	0.381	0.600
Training	0.003	0.784	0.06	0.672	0.055	0.267
RS × TS	0.138	0.436	0.41	0.914	0.730	0.769

Training system had a significant main-factor effect on *P. penetrans* per kg soil (*p* = 0.003) in British Columbia, with greater population densities under the Upright Fruiting Offshoots training system than under the other two systems which did not differ from each other (Table 1). Training system had a similar but marginal effect on *P. penetrans* in roots and soil combined (*p* = 0.06), but no effect on *P. penetrans* per g root (*p* = 0.78). In Nova Scotia, the effect of training system was marginally significant for *P. penetrans* per g root (*p* = 0.055) but with the Tall Spindle Axe training system supporting greater *P. penetrans* per g root than Upright Fruiting Offshoots or Kym Greene Bush training systems.

**Figure 2:**
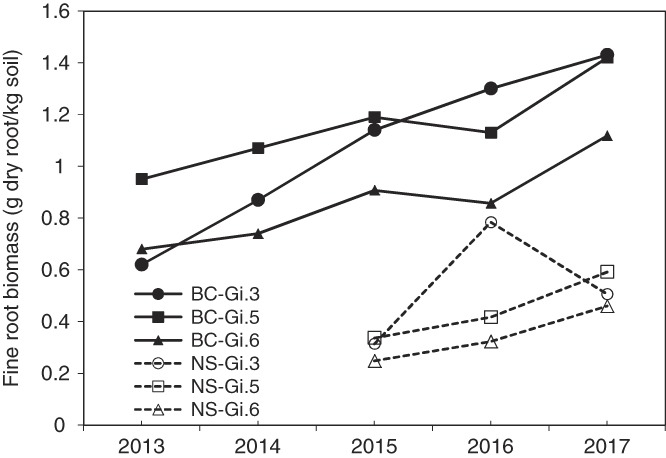
Temporal dynamics of the biomass of fine roots (<2 mm diameter) (g dry root/kg dry soil) under Gi.3, Gi.5, and Gi.6 trees during the course of the study, at the Summerland, British Columbia (BC, solid lines) and Kentville, Nova Scotia (NS, dashed lines) sites.

Fine root biomass was overall greater at the British Columbia site than the Nova Scotia site (Fig. 2). At the British Columbia site, there was a significant main-factor effect of rootstock with mean biomass values of 1.07, 1.15, and 0.86 g dry roots/kg soil for Gi.3, Gi.5, and Gi.6, respectively. At the Nova Scotia site, there was a significant sample date × rootstock interaction and main-factor effect of rootstock (*p* = 0.04 and 0.04, respectively) with mean biomass values of 0.53, 0.45, and 0.34 g dry roots/kg soil for Gi.3, Gi.5, and Gi.6, respectively.

### Effects of *P. penetrans* on tree growth parameters

Overall tree growth (trunk cross-sectional areas) was in the order Gi.6 > Gi.5 > Gi.3 at both sites. Overall, trees at the British Columbia site were substantially larger than at the Nova Scotia site, with 2017 overall average trunk cross-sectional areas of 78 vs 41 cm^2^, respectively.

**Figure 3:**
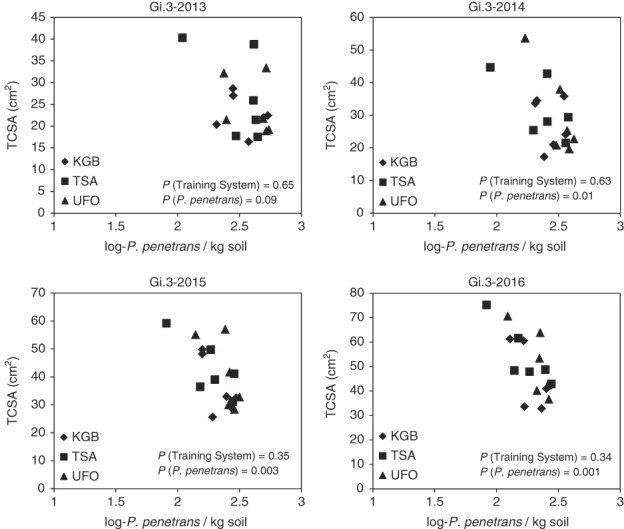
Relationships between running average *Pratylenchus penetrans* population densities and trunk cross-sectional areas (TCSA) for three different cherry tree training systems on Gisela 3 rootstock from 2013 through 2016 at the Summerland, British Columbia site. Training systems: KGB, Kym Greene Bush; TSA, Tall Spindle Axe; UFO, Upright Fruiting Offshoots.

**Figure 4:**
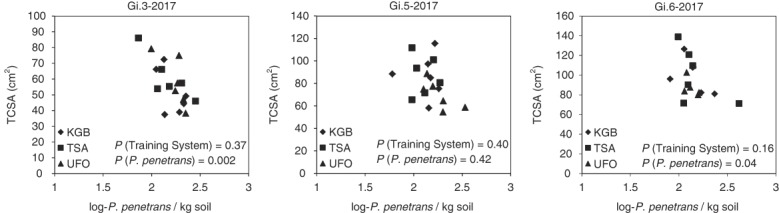
Relationships between running average *Pratylenchus penetrans* population densities and trunk cross-sectional areas (TCSA) for three different cherry training systems on Gisela 3 (left), Gisela 5 (center), and Gisela 6 (right) rootstocks at the end of 2017 at the Summerland, British Columbia site. Training systems: KGB, Kym Greene Bush; TSA, Tall Spindle Axe; UFO, Upright Fruiting Offshoot.

At the British Columbia site, when rootstock and training system were considered as fixed factors and running average *P. penetrans* population densities were considered as covariate in the ANCOVA model, there was a significant inverse relationship between running average *P. penetrans* population densities and trunk cross-sectional area that differed among rootstocks (rootstock × *P. penetrans* interaction *p* = 0.01). For Gi.3 trees, this inverse relationship between running average *P. penetrans* population densities and trunk cross-sectional area was significant in all years except 2013 (Fig. 3); for Gi.5 trees the relationship was not significant in any year (data not shown); and for Gi.6 trees there was a significant inverse relationship at the end of the experiment, in 2017 (Fig. 4). Training system did not have significant main-factor or interaction effects with *P. penetrans* (Figs. 3,4). Using normal yearly *P. penetrans* population data as covariate indicated significant inverse relationships between *P. penetrans* population densities and trunk cross-sectional diameter for three year × rootstock combinations: Gi.3 and Gi.5 in 2013 and Gi.3 in 2014 (data not shown).

Similar ANCOVA analyses of fine root biomass and total root biomass revealed few significant results. Out of the 30 combinations of year × rootstock × root parameter (fine roots, total roots) evaluated in British Columbia, there were only two significant relationships with *P. penetrans* population data, both of which were positive (total root biomass of Gi.5 in 2014; fine root biomass of Gi.6 in 2014). Out of the 18 combinations of year × rootstock × root parameter evaluated in Nova Scotia, there was only one significant negative relationship (total root biomass of Gi.3 in 2015). ANCOVA analyses of fruit yield and water relations parameters did not reveal statistically significant effects of *P. penetrans* (data not shown).

## Discussion

### Effects of rootstocks and training systems on *P. penetrans* population densities

In this experimental system, we assume that because each site was fumigated immediately before planting, differences in host suitability of the rootstocks should be manifest as differential buildup of *P. penetrans* populations in root tissues and root-zone soil within plots planted with the different rootstocks. We did not detect any consistent significant differences in *P. penetrans* population densities among the three rootstocks at either of the two sites, indicating that these rootstocks do not differ in their status as hosts for these two *P. penetrans* populations. There was a tendency for Gi.3 to support greater numbers of *P. penetrans* per g root than Gi.5, with Gi.6 being intermediate at the British Columbia site, which is consistent with earlier reports of analyses of individual sample dates in 2013 and 2014 (Neilsen et al., 2016; Reith et al., 2016). The differences observed in 2013 and 2014 did not persist through the subsequent years of sampling, however, and consequently repeated measures analyses of the entire five-year data set did not reveal any significant rootstock × sample date interactions or rootstock main-factor effects on *P. penetrans* per g root or any other nematode population parameters. Population densities were overall greater in 2013 and 2014 than in subsequent years at the British Columbia site. As discussed below, the decline in population densities at the British Columbia site was likely due to factors other than tree resistance to *P. penetrans*. We speculate that if a change in environmental conditions allowed for greater *P. penetrans* population buildup, differential resistance of the rootstocks could become evident again in the future.

The Upright Fruiting Offshoots training system resulted in greater soil population densities than the other training systems. The Upright Fruiting Offshoots system architecture has a narrower tree canopy than other training systems, resulting in the tree-row herbicide strip being exposed to more direct sunlight. Actual soil temperatures were not measured and compared between the training systems, but we speculate that the Upright Fruiting Offshoots system allowed for warmer growing season soil temperatures and perhaps greater weed growth, either of which could have fostered greater soil populations of *P. penetrans* relative to the other training systems. Such effects may not have been evident at the Nova Scotia site given overall different growing conditions, tree-row management practices, and smaller overall tree size.

### Relationships between *P. penetrans* population densities and tree growth

Although the rootstocks did not differ clearly in their host suitability, they did appear to differ in their tolerance to parasitism by *P. penetrans*. There was an inverse relationship between *P. penetrans* population densities and trunk cross-sectional area for Gi.3 trees in all years except 2013 and for Gi.6 trees in 2017 at the British Columbia site. Our data thus provide field evidence of the degree of impact of *P. penetrans* on cherry tree growth over multiple years, and indicate that Gi.3 rootstock is less tolerant than Gi.5 and Gi.6 rootstocks and should be avoided when replanting into *P. penetrans*-infested sites.

The impacts of nematode feeding on established fruit trees are likely to be cumulative, as nematode damage occurring in one year affects bud set and storage of carbohydrates and other resources, thereby affecting growth and yield in subsequent years (Schreiner et al., 2012). Such cumulative effects underlie the difficulty in detecting relationships between plant-parasitic nematode population densities and growth of established trees under field conditions when using nematode population data from the same year of tree measurement. To address this, we analyzed relationships between *P. penetrans* population densities and tree growth using population data that were calculated as a running average (by plot) to reflect multi-year cumulative nematode impacts, as well as using regular annual *P. penetrans* population data. We found that analyses using the running average data revealed more and stronger negative correlations across the 15 combinations of rootstocks and years than analyses of regular annual population data.

Reasons for the lack of an inverse relationship between *P. penetrans* population densities and tree growth at the Nova Scotia site are unclear, but we speculate that they are likely to be statistical rather than biological in nature. The Nova Scotia site had overall greater *P. penetrans* population densities and smaller trees than the British Columbia site. In addition, there was greater tree-to-tree variation at the Nova Scotia site, with average (across rootstocks) coefficients of variation in trunk cross-sectional area measurements of 23 and 40% for British Columbia and Nova Scotia, respectively, in 2017. It seems likely, therefore, that lower intensity of sampling (3 dates in Nova Scotia vs 10 in British Columbia) relative to inherent variability at the Nova Scotia site largely obscured any underlying relationship between *P. penetrans* abundance and rootstock growth. Another possibility is that the effect of *P. penetrans* on tree growth reaches a plateau above some threshold population density, such that when most plots are above the threshold, variation in tree growth is driven by factors other than *P. penetrans* population densities. Finally, other factors unique to the Nova Scotia site, such as more variable soil moisture regimes due to lack of irrigation, could have had over-riding effects on tree growth, thus obscuring any underlying relationships between *P. penetrans* abundance and rootstock growth at the Nova Scotia site.

*Pratylenchus penetrans* has been proven to parasitize a wide array of woody perennial fruit crops in the rosaceae including apple, pear, and other *Prunus* species such as sour cherry, peach, and plum in addition to sweet cherry. However, most studies of its impacts have been limited to relatively short-term experiments with very young (e.g. non-fruit bearing) trees in greenhouse pots, which are amenable to manipulation of pre-plant inoculum densities; few previous studies have linked *P. penetrans* population densities to vegetative growth or fruit production of established, fruit bearing perennial fruit crops under field conditions. The lack of data relating *P. penetrans* population densities to vigor or fruit yields of established trees makes it difficult for growers to assess the importance of *P. penetrans* to productivity of mature orchards and to make decisions about whether to implement nematode control practices. Notable previous studies on established trees include Santo and Wilson (1990) who used fenamiphos to experimentally suppress *P. penetrans* population densities in a block of Granny Smith apple on M7a rootstock and measured corresponding increases in yields. Similarly, Ferris et al. (2004) reported that oxamyl and fenamiphos suppressed early population buildup of *Mesocriconema xenoplax*, which also parasitizes sweet cherry, resulting in reduced cumulative ‘nematode dosage’ over six years and improved indices of tree vigor and reduced incidence of bacterial canker of Halford peach. Additional research using an approach similar to the ones used in Ferris et al’s. (2004) study and in our study, relating indices of cumulative nematode population pressure to growth or yields over multiple years, could drastically improve current understanding of the impacts of *P. penetrans* and other nematodes, such as *M. xenoplax*, on fruit trees.

### Between-site differences in population dynamics

Population densities of *P. penetrans* at the Nova Scotia site were consistently greater than at the British Columbia site and increased through the three years of sampling whereas they tended to decline slightly at the British Columbia site. Taken in isolation, the British Columbia data might suggest that these rootstocks became less suitable hosts with time. Such ontogenetic resistance to nematodes has been noted previously in *Prunus* germplasm (Fernández et al., 1995). Melakeberhan et al. (1994, 1997) studied *P. penetrans* population and plant growth responses of Gi.6 rootstock seedlings over three months after inoculation with nematode population densities of 625 to 1,300 *P. penetrans*/100 ml soil in greenhouse pots. Under these conditions of extraordinarily high initial inoculum levels and relatively short experiment duration, final *P. penetrans* population densities did not exceed initial population densities, leaving the actual host-status of the Gi.6 rootstock unclear in that study. It is noteworthy that population densities at the Nova Scotia site were more similar to those in the study of Melakeberhan et al. (1994, 1997) than the British Columbia site. Considering that population densities increased through time at one of the two sites in our study, and that we consistently recovered appreciable populations of *P. penetrans* from root tissue at both sites, we confirm that these rootstocks are in-fact hosts for *P. penetrans.*

The differences in overall population densities between the two sites could have been the result of different sample times, *P. penetrans* population genotypes, or different environmental conditions including levels of antagonists residing in the soil. Previous research in British Columbia has shown that *P. penetrans* population densities tend to be lowest in early- to mid-summer and peak in autumn (Vrain et al., 1996; Forge et al., 2016). The Nova Scotia site was sampled in October of each year while the British Columbia site was sampled in June (pre-harvest) and August (post-harvest) of each year, with the exception of 2013 when the first sample date was in April. The British Columbia sample dates were chosen to represent critical periods of tree growth, when we speculated that the *P. penetrans* would be having strongest effects on plant growth, rather than when the *P. penetrans* populations were at their peak. October was chosen as the sample time in Kentville to target peak population levels where no prior data were available.

Regarding potential differences in inherent aggressiveness of the two populations of *P. penetrans*, both populations were identified as *P. penetrans* via morphological and molecular characteristics (NCBI accession numbers MK176321 and MK282740 for British Columbia and Nova Scotia populations, respectively). However, differences among *P. penetrans* populations with respect to reproductive potential on a particular host have been noted previously (reviewed in Castillo and Vovlas, 2007), and the small region of 26S rDNA we used for species confirmation (D3A-D3B primers, Al-Banna et al., 1997, 2004) would not likely reveal such intra-specific differences. Future research comparing the responses of different *P. penetrans* populations on the same rootstocks and under the same environmental conditions would reveal whether there are inherent differences in aggressiveness between the Nova Scotia and British Columbia populations.

Differences in environmental conditions are not likely causes of the differences among the sites. Irrigation at the British Columbia site maintained relatively stable and moderate soil moisture contents optimal for tree growth during the growing season, which would also likely be optimal for nematode activity. Consequently, it seems unlikely that either excessive or inadequate soil moisture can explain the apparent decline in population densities at the British Columbia site. The Nova Scotia site was also generally cooler than the British Columbia site, accumulating approximately 20% fewer growing degree-days each year on average (2491 DD vs 1908 DD for British Columbia vs Nova Scotia, respectively) during the five years of the study, and there were no major differences between the two sites in minimum winter temperatures during the study period.

## Conclusion

In summary, our data indicate that these three Gisela-series rootstocks do not differ substantially in their suitability as hosts for *P. penetrans*. However, they do appear to differ in their tolerance to *P. penetrans*, with inverse relationships between *P. penetrans* population densities and trunk cross-sectional areas observed for Gi.3 in four of five years, and Gi.6 in the last year of the study at the British Columbia site. This relationship was not observed at the Nova Scotia site where *P. penetrans* population densities were overall much greater, sampling was less extensive and there was greater inherent variability in tree size. These data thus also provide quantitative field evidence of the potential impact of *P. penetrans* on cherry tree growth under British Columbia growing conditions, and suggest that Gi.3 rootstock should be avoided when planting sweet cherry into *P. penetrans*-infested sites.
